# ChlVPP alternating with PABlOE is superior to PABlOE alone in the initial treatment of advanced Hodgkin's disease: results of a British National Lymphoma Investigation/Central Lymphoma Group randomized controlled trial

**DOI:** 10.1054/bjoc.2001.1778

**Published:** 2001-05

**Authors:** B W Hancock, W M Gregory, M H Cullen, G Vaughan Hudson, A Burton, P Selby, K A Maclennan, A Jack, E M Bessell, P Smith, D C Linch, on behalf of the British National Lymphoma Investigation and Central Lymphoma Group

**Affiliations:** YCR Section of Clinical Oncology, Weston Park Hospital, Whitham Road, Sheffield, S10 2SJ; BNLI, MacDonald Buchanan Building, The Middlesex Hospital, Mortimer Street, London, WIN 8AA; Queen Elizabeth Hospital, Edgbaston, Birmingham, B15 2TH; CRC Trials Unit, Institute for Cancer Studies, University of Birmingham, Edgbaston, Birmingham, B15 2TH; St James's Hospital, Beckett Street, Leeds, LS9 7TF; The General Infirmary, Great George Street, Leeds, LS1 3EX; Nottingham City Hospital, Hucknall Road, Nottingham, NG5 1PB, UK

**Keywords:** alternating chemotherapy, ChlVPP/PABlOE, advanced Hodgkin's disease

## Abstract

The purpose of this randomized trial was to compare the efficacy of 6 cycles of prednisolone, Adriamycin (doxorubicin), bleomycin, vincristine (Oncovin) and etoposide (PABlOE) with 3 cycles of PABIOE that alternate with 3 cycles of chlorambucil, vinblastine, procarbazine and prednisone (ChlVPP) in patients with advanced Hodgkin's disease. Between October 1992 and April 1996, 679 patients were entered onto the study. 41 of these did not match the protocol requirements on review and were excluded from further analysis, most of these being reclassified as NHL on histological review. Of the remaining 638 patients, 319 were allocated to receive PABIOE and 319 were allocated to receive ChlVPP/PABlOE. The complete remission (CR) rates were 78% and 64%, for ChlVPP/PABlOE and PABIOE respectively after initial chemotherapy (*P*< 0.0001). 124 patients were re-evaluated subsequently following radiotherapy to residual masses. The CR rates changed from 78% to 88% for ChlVPP/PABlOE and from 64% to 77% for PABlOE when re-evaluated in this manner (treatment difference still significant *P* = 0.0002). The treatment associated mortality in the PABlOE arm was 2.2% (7 deaths), while there were no such deaths in the ChlVPP/PABlOE arm (*P* = 0.015). The failure-free survival was significantly greater in the ChlVPP/PABlOE arm (*P*< 0.0001) as was the overall survival (*P* = 0.01). The failure-free and overall survival rates at 3 years were 77% and 91% in the ChlVPP/PABlOE arm, compared with 58% and 85% in the PABIOE arm, respectively. These results indicate that ChlVPP alternating with PABIOE is superior to PABIOE alone as initial treatment for advanced Hodgkin's disease.© 2001 Cancer Research Campaign www.bjcancer.com


*British Journal of Cancer* (2001) **84**(10), 1293–1300
© 2001 Cancer Research Campaign
doi: 10.1054/ bjoc.2001.1778, available online at http://www.idealibrary.com on IDEAL®

http://www.bjcancer.com

### ChlVPP alternating with PABlOE is superior to PABIOE alone in the initial treatment of advanced Hodgkin's disease: results of a British National Lymphoma Investigation/Central Lymphoma Group randomized controlled trial

**BW Hancock[1], WM Gregory[2], MH Cullen[3], G Vaughan Hudson[2], A Burton[4], P Selby[5], KA Maclennan[5], A Jack[6], EM Bessell[7], P Smith[2] and DC Linch[2] on behalf of the British National Lymphoma Investigation and Central Lymphoma Group**

[1]YCR Section of Clinical Oncology, Weston Park Hospital, Whitham Road, Sheffield, S10 2SJ; [2]BNLI, MacDonald Buchanan Building, The Middlesex Hospital, Mortimer Street, London, WIN 8AA; [3]Queen Elizabeth Hospital, Edgbaston, Birmingham, B15 2TH; [4]CRC Trials Unit, Institute for Cancer Studies, University of Birmingham, Edgbaston, Birmingham, B15 2TH; [5]St James's Hospital, Beckett Street, Leeds, LS9 7TF; [6]The General Infirmary, Great George Street, Leeds, LS1 3EX; [7]Nottingham City Hospital, Hucknall Road, Nottingham, NG5 1PB, UK

**Summary** The purpose of this randomized trial was to compare the efficacy of 6 cycles of prednisolone, Adriamycin (doxorubicin), bleomycin, vincristine (Oncovin) and etoposide (PABlOE) with 3 cycles of PABIOE that alternate with 3 cycles of chlorambucil, vinblastine, procarbazine and prednisone (ChlVPP) in patients with advanced Hodgkin's disease. Between October 1992 and April 1996, 679 patients were entered onto the study. 41 of these did not match the protocol requirements on review and were excluded from further analysis, most of these being reclassified as NHL on histological review. Of the remaining 638 patients, 319 were allocated to receive PABIOE and 319 were allocated to receive ChlVPP/PABlOE. The complete remission (CR) rates were 78% and 64%, for ChlVPP/PABlOE and PABIOE respectively after initial chemotherapy ($P < 0.0001$). 124 patients were re-evaluated subsequently following radiotherapy to residual masses. The CR rates changed from 78% to 88% for ChlVPP/PABlOE and from 64% to 77% for PABlOE when re-evaluated in this manner (treatment difference still significant, $P = 0.0002$). The treatment associated mortality in the PABIOE arm was 2.2% (7 deaths), while there were no such deaths in the ChlVPP/PABlOE arm ($P = 0.015$). The failure-free survival was significantly greater in the ChlVPP/PABlOE arm ($P < 0.0001$) as was the overall survival ($P = 0.01$). The failure-free and overall survival rates at 3 years were 77% and 91% in the ChlVPP/PABlOE arm, compared with 58% and 85% in the PABIOE arm, respectively. These results indicate that ChlVPP alternating with PABIOE is superior to PABIOE alone as initial treatment for advanced Hodgkin's disease. © 2001 Cancer Research Campaign http://www.bjcancer.com

**Keywords**: alternating chemotherapy; ChlVPP/PABlOE, advanced Hodgkin's disease

It is now nearly 30 years since cyclical combination chemotherapy with MO(orV)PP (mustine (mechlorethamine), vincristine (Oncovin) or vinblastine (Velbe), procarbazine and prednisolone) revolutionized the management of advanced Hodgkin's disease (Devita et al, 1970; Nicholson et al, 1970). Long-term survival was observed in approximately half of the patients treated with these regimens (Longo et al, 1986; Linch and Vaughan Hudson, 1988).

The Milan group developed an Adriamycin containing regimen, ABVD (Adriamycin (doxorubicin), bleomycin, vinblastine, dacarbazine), (Santoro et al, 1982) and alternated this with MOPP, in accordance with the Goldie-Coldman hypothesis (Goldie et al, 1982). Their trial comparing MOPP/ABVD with MOPP alone showed the alternating approach to be superior. However, other studies comparing single with alternating regimens produced conflicting results (Gans et al, 1982; Vinciguerra et al, 1986; Longo et al, 1991; Canellos et al, 1992).

*Received 20 June 2000*
*Revised 11 January 2001*
*Accepted 20 February 2001*

*Correspondence to:* BW Hancock

Also the Cancer and Leukaemia Group B (CALGB) trial (where ABVD and MOPP/ABVD were equally efficacious and both better than MOPP) implied that any improvements in treatment results may have been due simply to the use of a more active doxorubicin-containing regimen, rather than a consequence of alternating combinations (Canellos et al, 1992). The importance of this question was reinforced by increasing evidence of permanent infertility in males and second malignancies in patients treated with alkylating agents and procarbazine.

Other groups have substituted chlorambucil for mustine in an attempt to increase patient acceptability, and hence compliance, by reducing the toxicity of chemotherapy whilst maintaining efficacy. The resulting ChlVPP regimen appeared to fulfil this aim (Selby et al, 1980). The British National Lymphoma Investigation (BNLI) confirmed this in a randomized study of 299 patients where LOPP (L for Leukeran (chlorambucil)) was compared with MOPP (Hancock et al, 1991). The trend towards enhanced acceptability continued with a study by the Central Lymphoma Group (CLG), in which the ABVD component in alternating therapy was modified by replacing dacarbazine with etoposide, adding prednisolone, and switching vinca alkaloids to form PABIOE (prednisolone, Adriamycin (doxorubicin), bleomycin, Oncovin (vincristine), etoposide), which was alternated with ChlVPP (Cullen et al, 1994). In 216 patients with advanced Hodgkin's disease treated with this regimen the 5-year survival was 78%. At the same time the BNLI showed that LOPP alternating with EVAP (etoposide, vinblastine (Velbe), Adriamycin (doxorubicin), prednisolone) was superior to LOPP alone in a randomized trial including 594 patients (Hancock et al, 1992).

In view of the similarity of PABIOE to ABVD and the favourable results for ABVD in the randomized (albeit small) CALGB (Canellos et al, 1992) the BNLI and CLG combined to compare the effective ChlVPP/PABIOE regimen with PABIOE alone in a randomized, multicentre trial in advanced HD.

### PATIENTS AND METHODS

### Patients

The criteria for inclusion were as follows: stage I or II disease with either bulky disease or 'B' symptoms or stage III or IV disease; age 15 years to 69 years; opportunity for adequate long-term follow-up must have been anticipated; freedom from any other known serious disease that might limit severely the patient's life expectancy; no previous chemotherapy and/or radiotherapy (RT) except as an emergency measure for obstructive symptoms; surgical staging was not required, but all patients were required to have either lymphangiography or computed tomographic (CT) scanning of the abdomen; histopathologic diagnosis confirmed by the BNLI/CLG histopathology panel; and informed consent obtained. Ethics committee approval for the study was obtained at all anticipating centres.

Patients were randomized to receive either PABIOE or ChlVPP alternating with PABIOE. Randomization was performed by a telephone call to one of two central offices using cards within sealed envelopes without stratification. Staging was performed according to the Ann Arbor criteria (Carbone et al, 1971) and histologic grading according to previously published BNLI criteria (Bennett et al, 1989).

### Treatment

Chemotherapy regimen doses and scheduling are listed in Table 1. Patients were completely reassessed after 3 cycles of chemotherapy. If the patient was in clinical CR at this time, 3 additional cycles of chemotherapy were scheduled (i.e. a total of 6 cycles). If after 3 cycles the assessment showed the patient to be progressively improving, then the treatment was continued (provided that progressive improvement persisted) until a clinical CR was attained, after which 2 more cycles of chemotherapy were scheduled (i.e. a total of 8 cycles for a patient in clinical CR after the sixth cycle). The maximum number of 8 cycles was stipulated. If there was progressive disease, no progressive improvement or disease relapse then treatment was changed. Salvage therapy was as the local clinician's discretion.

Patients who had a CR with chemotherapy were eligible for further randomization to radiotherapy (35 Gy in 20 fractions in 4 weeks) to areas of bulk disease (>5 cm) or no radiotherapy. Involved field radiotherapy was used such that the disease was encompassed in one treatment volume. The use of extended field radiotherapy to cover sites which were either not involved with HD initially or contained non-bulky HD was not recommended to avoid excessive bone marrow irradiation. The routine use of involved field radiotherapy after CR with chemotherapy was also not recommended. Patients who had a PR with chemotherapy could receive involved field radiotherapy (35–40 Gy in 20 fractions in 4 weeks) to residual masses.

Of the 642 patients randomized to either of the chemotherapy regimens, 208 patients (32%) received radiotherapy after completion of chemotherapy (ChlVPP/PABIOE, 107 patients, PABIOE, 101 patients) (Table 2).

61 patients in CR after chemotherapy were randomized between radiotherapy (32 patients) and no radiotherapy (29 patients). 3 patients randomized to radiotherapy did not receive it (refusal, 2 patients; disease progression, 1 patient). Thus 179 patients received radiotherapy after chemotherapy other than the 29 patients in the radiotherapy trial. Of these 179 patients, 54 patients were in CR after chemotherapy and 124 patients were in PR (119 patients) or NR (5 patients). In one patient the remission status was unknown.

Clinical response was determined by repeating initially abnormal investigations. Investigation of persisting equivocal abnormalities was at the discretion of the clinician; CRu (uncertain) was not recorded.

### Statistical methods

The main endpoint of the study was survival. Secondary endpoints were failure-free survival and achievement of CR. The trial was set up with an intention to recruit 700 patients. This would enable

**Table 1** Regimen doses

| | |
|---|---|
| **PA(BI)OE regimen** | |
| Prednisolone | 40 mg/m² (maximum 60 mg) p.o. daily for 10 days |
| Adriamycin (doxorubicin) | 40 mg/m² iv day 1 |
| Bleomycin | 10 units/m² days 1 and 8 *for first 4 cycles only* |
| Vincristine | 1.4 mg/m² (maximum 2 mg) i.v. Days 1 and 8 |
| Etoposide | 200 mg/m² p.o. daily for 3 days[a] |
| Cycle repeated every 21 days maximum 8 cycles | |
| **ChlVPP/PABIOE regimen** | |
| Chlorambucil | 6 mg/m² (maximum 10 mg) p.o. daily days 1–14 |
| Vinblastine | 6 mg/m² (maximum 10 mg) i.v. days 1 and 8 |
| Procarbazine | 100 mg/m² (maximum 200 mg) p.o. daily days 1–14 |
| Prednisolone | 40 mg/m² (maximum 60 mg) p.o. daily days 1–14 |

The alternation of ChlVPP/PABIOE consists of initial treatment with a cycle of ChIVPP for 2 weeks followed by a 2 week gap with no chemotherapy. At the beginning of the 5th week a cycle of PABIOE is given and lasts for 10 days. There is an 11 day gap without chemotherapy and then the next cycle of ChlVPP begins (maximum 8 cycles 4 ChlVPP, 4PABIOE).

[a]If nadir (day 10–14) WBC <1.0 × 10⁹ l⁻¹ decrease etoposide to 2 days. If nadir WBC > 1.5 × 10⁹ l⁻¹ increase etoposide to 4 days.

 *© 2001 Cancer Research Campaign*

**Table 2** Number of patients receiving radiotherapy

| Response to chemotherapy | ChlVPP/PABIOE No. receiving RT | PABIOE No. receiving RT |
|---|---|---|
| CR | 52 | 31 |
| PR | 52 | 67 |
| NR | 2 | 3 |
| Unknown | 1 | 0 |
| Total | 107 | 101 |

the detection of a 10% difference in survival at 5 years with a 5% chance of a false positive result and a 10% chance of a false negative result. Survival was calculated as the time from randomization to death, or to the date of last follow-up if the patient was still alive. The data included follow-up until June 1998. CR was defined as complete disappearance of all disease for a minimum of 1 month after the completion of therapy. PR was defined as the disappearance of at least 50% of known disease. CR rates were compared in the 2 arms of the trial by use of Fisher's Exact Test where possible and otherwise by a chi-squared ($\chi^2$) test with Yates' correction.

Survival curves were calculated by the method of Kaplan and Meier (1958) and statistical comparison of curves was performed by the log-rank test as described by Peto et al (1977). The hazard ratio, with associated confidence limits (Altman, 1991), was used to quantify the increased risk associated with one treatment compared to the other. This assumes proportional hazards throughout the time period of the study, and enables a single measure to be used to quantify any treatment difference. Failure-free survival was recorded as the time to progression for complete and partial responders, and time to treatment failure for non-responders. 2 patients (one in each arm) who received high-dose chemotherapy (HDC) in partial remission without having relapsed were censored at the date of HDC when calculating failure-free survival. This was done to avoid bias since this was an additional non-protocol treatment.

Factors affecting achievement of complete remission were analysed using multivariate logistic regression methods. Cox's multivariate proportional hazards model (Cox, 1972) was used to evaluate prognostic factors for failure-free and overall survival. For these multivariate analyses some variables had a few missing values. To enable inclusion of all the patients in the analyses these missing values were estimated (imputed) by linear regression methods using values from other available variables. The analyses were also undertaken without the imputed values, to confirm the results, which in all cases were nearly identical in magnitude. A significance level of 0.05 was used for inclusion in all the multivariate models.

Continuous factors such as age, albumin and WBC were grouped categorically with different cut-off points and compared with the results when analysed continuously, in order to determine sensible prognostic groups as suggested by Wagstaff et al (Wagstoff et al, 1988). Cut-offs used by others were considered where possible. For instance, Dhaliwal et al (Dhaliwal et al, 1993) analysed albumin in 3 subgroups, namely ≥ 32, 33–39 and ≤ 40. We combined adjacent equivalent groups, thus finding, in this case for albumin, that very low albumin values (≤ 32) predicted for poor response while higher values (≥ 40) predicted for better failure-free survival.

### RESULTS

### Patients

A total of 679 patients were entered onto this trial between October 1992 and April 1996. The trial was prematurely closed when interm analysis of data available as of January 1996 showed a significant difference between the arms of the study. Patients still on study receiving PABIOE were converted to ChlVPP/PABIOE.

### Exclusions

Of the 679 patients randomized 41 (6%) were excluded from analysis. Reasons for exclusion were: 21 incorrect histology on review, 4 too old, 6 previously treated, 1 previous cancer, 3 treatment protocol violations and 6 converted to ChlVPP/PABIOE after trial stopped.

### Patient and chemotherapy characteristics

The 2 arms of the trial were balanced for all patient characteristics. (Table 3). More patients on ChlVPP/PABIOE received 8 cycles of treatment but this was not statistically significant (Table 4).

### Response (Table 5)

Response was available in 313 of the 319 patients randomized to receive PABIOE, and 200 of these (64%) achieved CR. Of the 319 randomized to receive ChlVPP/PABIOE 247/315 (78%) achieved CR. The difference in complete response rate (78% vs 64%) was significant ( $\chi^2$ with Yates correction = 15.4, $P < 0.0001$). 124 patients were subsequently re-evaluated following radiotherapy to residual masses (ChlVPP/PABIOE: 55 patients, PABIOE: 69 patients). The CR rates changed to 88% (278/315) for ChlVPP/-PABIOE and to 77% (240/313) for PABIOE when re-evaluated in this manner (treatment difference still significant, $\chi^2$ with Yates correction = 13.8, $P = 0.0002$). 72 patients were converted from PR to CR with additional radiotherapy and one from NR to PR. The duration of response of these 72 patients is very similar to that of the complete responders after chemotherapy (data not shown).

The logistic regression analysis (see Table 6) showed that treatment with PABIOE ($P < 0.0001$, odds ratio 2.1), white blood count (WBC) $\geq 20 \times 10^9$ $l^{-1}$ ($P = 0.005$), albumin $\leq 32$ g $l^{-1}$ ($P = 0.01$) and being Nodular Sclerosis Grade II histology ($P = 0.02$) predicted for a worse proportion achieving complete response. There was no significant effect of age on complete response rate ($P = 0.34$).

38 of 66 (58%) relapsers in the ChlVPP/PABIOE arm subsequently needed high-dose therapy (with autologous peripheral blood stem cell or bone marrow transfusion) compared with 75/120 (63%) in the PABIOE arm ($P = 0.53$, Fisher's Exact test). So although twice as many patients in the PABIOE arm received high-dose therapy (HDT) the proportions in the 2 arms were similar. Follow-up after relapse is short in these patients to date so the simple proportion of patients receiving HDT is an underestimate of the eventual numbers likely to require it. Actuarially the proportions of patients receiving HDT are approximately 80% in both arms one year following relapse (data not shown).The survival of those patients requiring salvage therapy after failure of first line treatment was similar in both arms (data not shown).

© 2001 Cancer Research Campaign 

**Table 3** Patient characteristics by treatment

| | | ChlVPP/PABIOE | | PABIOE | |
|---|---|---|---|---|---|
| Total number of patients randomized | | 337 | | 342 | |
| Number of ineligible patients | | 18 | | 23 | |
| Total used in analysis | | 319 | (100%) | 319 | (100%) |
| Age (years) | 15–49 | 269 | (84%) | 268 | (84%) |
| | ≥ 50 | 50 | (16%) | 51 | (16%) |
| Gender | Male | 191 | (60%) | 191 | (60%) |
| | Female | 128 | (40%) | 128 | (40%) |
| Stage | I | 19 | (6%) | 32 | (10%) |
| | II | 156 | (49%) | 136 | (43%) |
| | III | 76 | (24%) | 89 | (28%) |
| | IV | 67 | (21%) | 61 | (19%) |
| | Not known | 1 | (1%) | 1 | (1%) |
| Symptoms | A | 112 | (35%) | 114 | (35%) |
| | B | 203 | (63%) | 203 | (64%) |
| | Not known | 4 | (1%) | 2 | (1%) |
| Histology | Mixed cellularity | 49 | (15%) | 56 | (18%) |
| | Lymphocyte depleted | 3 | (1%) | 2 | (1%) |
| | Nodular sclerosis (NS)I | 139 | (44%) | 132 | (41%) |
| | Nodular sclerosis (NS)II | 119 | (37%) | 125 | (39%) |
| | Not known | 9 | (3%) | 4 | (1%) |
| Centre | BNLI | 218 | (68%) | 215 | (67%) |
| | CLG | 101 | (32%) | 104 | (33%) |
| WBC (x $10^9$ $l^{-1}$) | <20 | 295 | (93%) | 292 | (92%) |
| | ≥20 | 12 | (4%) | 15 | (5%) |
| | Not known | 12 | (4%) | 12 | (4%) |
| Albumin (g $l^{-1}$) | <40 | 153 | (48%) | 145 | (45%) |
| | ≥40 | 143 | (45%) | 149 | (47%) |
| | Not known | 23 | (7%) | 25 | (8%) |

### **Failure free survival** (Figure 1)

There was a significant difference in the failure-free survival (FFS) between treatment groups ($\chi^2$ = 25.6, $P$ < 0.0001), with the hazard of failure for ChlVPP/PABIOE being approximately half that for PABIOE (50% ± 10%). At 3 years the FFS rates were 77% and 58% for ChlVPP/PABIOE and PABIOE, respectively. On multivariate analysis (see Table 7) the factors predicting for better FFS were treatment with ChlVPP/PABIOE ($\chi^2$ = 29.4, $P$ < 0.0001), stage (I + II better than III better than IV, $\chi^2$ = 14.0, $P$ = 0.0002), albumin ≥40 ($\chi^2$ = 6.7, $P$ = 0.01), WBC < 20 ($\chi^2$ = 6.1, $P$ = 0.01) and not having mixed cellularity histology ( $\chi^2$ = 4.8, $P$ = 0.03). The hazard for progressing on PABIOE compared to ChlVPP/PABIOE was virtually unchanged (2.01 changing to 2.12) after allowance for the other significant factors (stage, albumin and WBC).

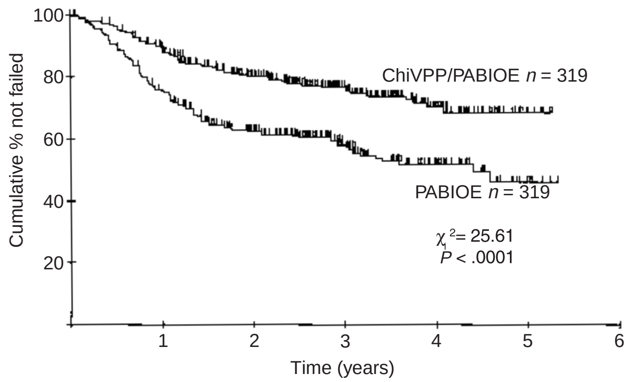

**Figure 1** Failure-free survival by treatment

### **Overall survival** (Figure 2)

The median duration of follow-up was 2.7 years (range 3 months to 5 1/2 years). There was a significant difference in overall survival between the 2 regimens ($\chi^2$ = 6.06, $P$ = 0.01). The hazard of death was increased by 43% ± 18% in the PABIOE arm compared to the ChlVPP/PABIOE arm. The magnitude of this effect is not dissimilar from that for failure-free survival (50% ± 10%, see Figure 1), although the $P$ value is clearly much less significant. This is because there are many more failures than deaths in the trial so far. At 3 years the survival rates were 91% and 85% for ChlVPP/PABIOE and PABIOE, respectively. Stage I patients fared slightly worse than stage II patients ($P$ = 0.04), probably because they had more bulky disease (however, only 51 (8%) stage I patients were included). Using Cox's proportional hazards model analysis (see Table 7) the factors predicting for better overall survival were age <50 years ($\chi^2$ = 24.6, $P$ < 0.0001), stage

**Table 4** Number of cycles received

| Cycle | ChlVPP/PABIOE | | PABIOE | |
|---|---|---|---|---|
| | *n* | % | *n* | % |
| 0 | 1 | (0) | 0 | (0) |
| 1 | 6 | (2) | 6 | (2) |
| 2 | 3 | (1) | 4 | (1) |
| 3 | 3 | (1) | 8 | (3) |
| 4 | 8 | (3) | 14 | (4) |
| 5 | 4 | (1) | 9 | (3) |
| 6 | 171 | (55) | 181 | (58) |
| 7 | 13 | (4) | 21 | (7) |
| 8 | 102 | (33) | 70 | (22) |

This information was unavailable in 14 patients; 8 on the ChlVPP/PABIOE arm and 6 on the PABIOE arm.

 *© 2001 Cancer Research Campaign*

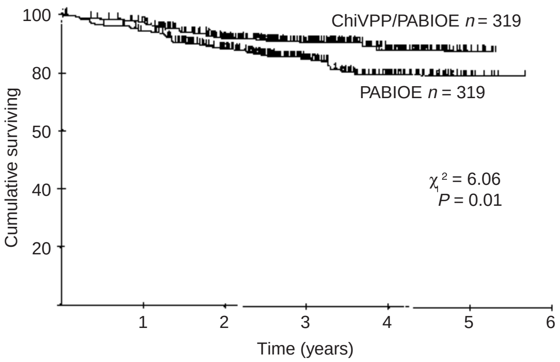

**Figure 2** Survival by treatment

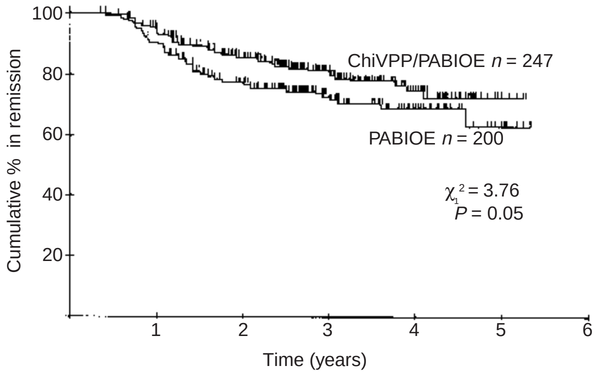

**Figure 3** Duration of complete remission by treatment

**Table 5** Responses after chemotherapy alone and after additional radiotherapy

| | ChlVPP/PABIOE | +RT | PABIOE | +RT |
|---|---|---|---|---|
| CR | 247/315 (78%) | 278/315 (88%) | 200/313 (64%) | 243/313 (78%) |
| PR | 58/315 (1/8%) | 25/315 (8%) | 93/313 (30%) | 53/313 (17%) |
| NR | 10/315 (3%) | 12/315 (4%) | 20/313 (6%) | 20/313 (6%) |

CR = Complete response; PR = Partial response; NR = Non response.

**Table 6** Multivariate logistic regression results for achievement of complete remission

| Variable | z[a] | *P* value | OR[b] | 95% CI[c] on OR[b] |
|---|---|---|---|---|
| Treatment | 4.0 | <.0001 | 2.07 | (1.45–2.98) |
| WBC (<20 v ≥20) | 2.8 | .005 | 3.20 | (1.42–7.25) |
| Albumin (≤32 v >32) | 2.5 | .01 | 1.90 | (1.16–3.12) |
| Histology (NSII v others) | 2.3 | .02 | 1.53 | (1.06–2.20) |

[a] Standardized normal deviate; [b] Odds ratio; [c] Confidence interval.

(I + II better than III better than IV, $\chi^2 = 9.3$, $P = 0.002$), WBC <20 ($\chi^2 = 5.5$, $P = 0.02$) and absence of B symptoms ($\chi^2 = 6.5$, $P = 0.01$). The significance of treatment remained unchanged ($\chi^2 = 6.8$, $P = 0.009$) in the multivariate analysis, having adjusted for the effects of these 4 factors.

### Duration of remission (Figure 3)

There was a significant difference in remission duration by treatment ($\chi^2 = 3.76$, $P = 0.05$) with a 32% ± 17% reduction in the hazard of relapse in the ChlVPP/PABIOE arm compared to the PABIOE arm. The number of events was, however, small.

### Toxicity (Table 8)

There were 7 early deaths that were probably related to treatment (septicaemia, with residual Hodgkin's disease in all except 2) in the PABIOE arm and no treatment-related deaths in the ChlVPP/-PABIOE arm ($P = 0.015$, Fisher's Exact Test).

Information was collected for haematological toxicity, infections and neuropathy. The data presented is the worst WHO toxicity score over the entire treatment period. Haematological toxicity data was available for 507 patients; data on infection was available in 500 patients and on neuropathy in 494 patients.

It is apparent that Ch1VPP/PABIOE results in more haematologic suppression ($\chi^2_1$ (trend) = 32.15, $P < 0.0001$). Though the incidence of infection was not different between the 2 treatments ($\chi^2_1$ (trend) = 0.96, $P = 0.33$), more neuropathy was seen in the PABIOE arm ($\chi^2_1$ (trend) = 6.5, $P = 0.01$). Other toxicities (gastrointestinal, skin, phlebitis, alopecia, effects of steroids) were not different between the 2 arms.

### Mortality (Table 9)

There have been 48 deaths in the PABIOE arm and 27 deaths in the ChlVPP/PABIOE arm. Death in both groups was related mostly to disseminated Hodgkin's disease. A total of 4 patients died from secondary malignancy (2 in each arm).

### DISCUSSION

The ChlVPP/PABIOE regimen is a modification of MOPP/ABVD with reduced subjective toxicity (substituting chlorambucil for mechlorethamine, and etoposide for dacarbazine) (Cullen et al, 1994). There are also scheduling changes resulting in an increase in anthracycline dose-intensity and a reduction in overall treatment duration. The CR rate for ChlVPP/PABIOE (plus radiotherapy where indicated) in the present randomized trial is 87% compared with 85% in the phase II study. The overall survival at 3 years is 91% compared with 78% at 5 years in phase II. The efficacy of this regimen has clearly been maintained in the transition from phase II (in 216 patients) to phase III (in 319 patients).

During this same period ABVD was shown to be superior to MOPP and possibly equivalent to MOPP/ABVD, although with only 115 to 123 patients per arm the CALGB trial was not powered to detect small, perhaps clinically worthwhile differences (Canellos et al, 1992). More recently ABVD was reported to be equivalent to MOPP/ABV (Duggan et al, 1997). Consequently

*© 2001 Cancer Research Campaign*

**Table 7** Cox's proportional hazards regression model results

**1. Factors predicting for better failure-free survival**

| Variable | $\chi^2$ | *P* value | HR* | 95% CI on HR[a] |
|---|---|---|---|---|
| Treatment with ChlVPP/PABIOE | 29.4 | <.0001 | 2.15 | (1.62–2.86) |
| Stage (I+II>III>IV) | 14.0 | .0002 | 1.38 | (1.17–1.64) |
| Albumin ≥ 40 | 6.7 | .01 | 1.45 | (1.09–1.92) |
| WBC < 20 | 6.1 | .01 | 2.00 | (1.21–3.31) |
| Histology other than MC | 4.8 | .03 | 1.56 | (1.02–2.38) |

**2. Factors predicting for better survival**

| Variable | $\chi^2$ | *P* value | HR* | 95% CI on HR[a] |
|---|---|---|---|---|
| Age < 50 | 24.6 | <.0001 | 3.62 | (2.25–5.82) |
| Stage (I+II>III>IV) | 9.3 | .002 | 1.54 | (1.17–2.03) |
| Treatment with ChlVPP/PABIOE | 6.8 | .009 | 1.85 | (1.15–2.97) |
| Absence of B symptoms | 6.5 | .01 | 2.05 | (1.14–3.70) |
| WBC < 20 | 5.5 | .02 | 2.52 | (1.27–5.20) |

[a] Hazard ratio. The following additional factors were included in these analyses and found not to be significant: gender, histology, bone marrow involvement, haemoglobin, ESR, centre.

**Table 8** WHO toxicity grade by treatment

| | | WHO toxicity grade | | | | |
|---|---|---|---|---|---|---|
| **Toxicity** | **Treatment** | **0 (%)** | **1 (%)** | **2 (%)** | **3 (%)** | **4 (%)** |
| Haematological | ChlVPP/PABIOE | 53 (21) | 26 (10) | 53 (21) | 47 (19) | 72 (29) |
| | PABIOE | 91 (36) | 44 (18) | 49 (20) | 38 (15) | 28 (11) |
| Infections | ChlVPP/PABIOE | 117 (48) | 44 (18) | 44 (18) | 29 (12) | 10 (4) |
| | PABIOE | 123 (49) | 54 (22) | 41 (16) | 25 (10) | 8 (3) |
| Neuropathy | ChlVPP/PABIOE | 129 (55) | 84 (36) | 17 (7) | 5 (2) | 1 (0) |
| | PABIOE | 119 (47) | 83 (33) | 40 (16) | 9 (4) | 1 (0) |

**Table 9** Causes of death

| | Deaths(number) | |
|---|---|---|
| **Cause** | **ChlVPP/PABIOE** | **PABIOE** |
| Hodgkin's disease (HD) | 21 | 29 |
| Treatment related – HD present | 0 | 5 |
| Treatment related – without HD | 0 | 2 |
| Treatment related – following subsequent high dose therapy after relapse | 0 | 3 |
| Secondary leukaemia that caused death | 2 | 0 |
| Secondary solid cancer that caused death | 0 | 2 |
| Cardiac related | 1 | 2 |
| Suicide (with HD present) | 1 | 0 |
| Intercurrent disease – other cause | 0 | 2 |
| Unspecified | 2 | 3 |
| Total | 27 | 48 |

ABVD is widely used across the world, since the elimination of mechlorethamine and procarbazine offers distinct long-term toxicity advantages in preservation of fertility, and low incidence of second malignancies (Canellos, 1996).

The design of this trial comparing ChlVPP/PABIOE with non-alternating PABIOE followed naturally in an attempt to demonstrate similar equivalence of a less toxic regimen, including preservation of fertility. In the event, PABIOE is clearly inferior in efficacy, even after adjusting for other significant prognostic factors (stage, albumin, and white cell count for failure-free survival and stage, age, white cell count and B symptoms for overall survival). Further similar attempts to minimize subjective toxicity may be strictly limited.

We are unable to explain the inferiority of the PABIOE regimen, particularly in view of the favourable results for ABVD in the CALGB trial (Canellos et al, 1992). However, progression-free

 *© 2001 Cancer Research Campaign*

and overall survival for PABIOE is similar to that found for LOPP – the 4 drug regimen employed in the preceeding BNLI studies (Hancock et al, 1992) (updated but unpublished data).

The role of radiotherapy in advanced HD is still debated. Partial remission may be converted to CR, as demonstrated in our study, but in other situations (including previous bulk disease with complete remission after full-course chemotherapy) overall survival does not seem to be prolonged (Loeffler et al, 1998).

The results with ChlVPP/PABIOE are similar to those reported in multi-centre randomized trials incorporating doxorubicin-containing regimens. These include alternating schedules i.e. MOPP/ABVD (Bonadanna et al, 1986) LOPP/EVAP (Hancock et al, 1992), or 'hybrid' schedules i.e. ChlVPP/EVA (Radford et al, 1995), MOPP/ABV (Glick et al, 1998) which, in turn, produce superior FFS to MOPP, LOPP, MVPP and sequential MOPP/ABVD respectively. The devices of alternating different combinations or 'hybridizing' active regimens may not, in themselves, be as important as the inclusion of enough of the most effective drugs, in adequate dosage, in close enough time proximity. The most recent device is dose-intensification with growth factor support in regimens like Stanford V (Bartlett et al, 1995) and escalated BEACOPP (Diehl et al, 1997). It remains to be seen whether these are superior to the best non-intensified multi-drug regimens listed above. If so, they may represent over-treatment for many patients with advanced HD. ChlVPP/PABIOE is unique among the very effective regimens tested in a multicentre context in over 500 patients, in having no acute treatment-related mortality.

Finally, in those cases not requiring more intensive therapy (which may be the majority), we need to know whether ABVD is as good as the multi-drug regimens. To resolve this the UK Lymphoma Group is now well advanced in a major randomized comparison of ABVD with alternating ChlVPP/PABIOE or hybrid ChlVPP/EVA with individual physicians selecting their preferred multidrug regimen. The target accrual of 800 patients will be achieved by late 2001.

### ACKNOWLEDGEMENTS

The BNLI and CLG would like to thank the collaborators from the referring centres whose patients are included in this analysis. The trial was supported by the Cancer Research Campaign; the BNLI is also grateful for financial help from the Lymphoma Research Trust, the Lisa Lear Fund, the Isle of Man Anti-cancer Association and the Lymphoma Association.

### REFERENCES

Altman DG (1991) Analysis of survival times in "Practical Statistics for Medical Research' by Altman, DG, Chapman & Hall, London. pp. 383–384

Bartlett NL, Rosenberg SA, Hoppe RT, Hancock SL and Horning SJ (1995) Brief chemotherapy, Stanford V, and adjuvant radiotherapy for bulky or advanced-stage Hodgkin's disease: A preliminary report. *J Clin Oncol* **13**: 1080–1088

Bennett MH, MacLennan KA and Vaughan Hudson B (1989) The clinical and prognostic relevance of histopathological classification in Hodgkin's Disease. in Fenoglio-Preiser CM, Wolff M and Rilke F (ed): *Progress in Surgical Pathology* New York. Field and Wood. pp. 127–151

Bonadonna G, Valagussa P and Santoro A (1986) Alternating non-cross resistant combination chemotherapy or MOPP in stage IV Hodgkin's disease: A report of 8-year results. *Annal Intern Med* **104**: 739–46

Canellos GP (1996) Is ABVD the standard regimen for Hodgkin's disease based on randomised CALGB comparison of MOPP, ABVD and MOPP alternating with ABVD. *Leukaemia* **10 (**suppl.2**)**: S68

Canellos GP, Anderson JR, Propert KJ, Nissen N, Cooper MR, Henderson ES, Green MR, Gottlieb A and Peterson BA (1992) Chemotherapy of advanced Hodgkin's disease with MOPP, ABVD, or MOPP alternating with ABVD. *New Engl J Med* **327**: 1478–1484

Carbone PP, Kaplan HS, Musshoff N, Smithers DW and Tubiana M (1971) Report of the Committee on Hodgkin's Disease staging classifications. *Cancer Res* **31**: 1860–1861

Cox DR (1972) Regression models and life tables. *J Roy Statist Soc (B)* **34**: 187–220

Cullen MH, Stuart NSA, Woodroffe C, Murphy A, Fletcher J, Blackledge GRP, Child JA, Grieve RJ and Jones EL (1994) ChlVPP/PABIOE and radiotherapy in advanced Hodgkin's disease. *J Clini Oncol* **12**: 779–787

De Vita VT, Serpick AA and Carbone PP (1970) Combination chemotherapy in the treatment of advanced Hodgkin's disease. *Annal Intern Med* **73**: 881–895

Dhaliwal HS, Rohatiner AZS, Gregory W, Richards MA, Johnson PWM, Whelan JS, Gallagher CJ, Matthews J, Ganesan TS, Barnett MJ, Waxman JH, Stansfeld AG, Wrigley PFM, Slevin ML, Malpas JS and Lister TA (1993) Combination chemotherapy for intermediate and high grade non-Hodgkin's lymphoma. *Br J Cancer* **68**: 767–774

Diehl V, Sieber M, Ruffer U, Cathan B, Hasenclever D, Pfreundschuh M, Leoffler M, Lieberz D, Koch P, Adler M and Tesch H (1997) An intensified chemotherapy regimen in advanced Hodgkin's disease. *Annal Oncol* **8**: 143–148

Duggan D, Petroni G, Johnson J, Hanson K, Glick J, Connors JM, Cherny R, Barcos M and Peterson BA (1997) MOPP/ABV versus ABVD for advanced Hodgkin's disease – a preliminary report of CALGB 8952 (with SWOG, ECOG, NCIC). *Proceedings of American Society of Clinical Oncology* **16**: 12a (abstract 43)

Gams RA, Durant JR and Bartolucci AA (1982) Chemotherapy for advanced Hodgkin's disease: Conclusions from the Southeastern Cancer Study Group. *Cancer Treatment Reports* **66**: 899–905

Goldie J, Coldman A and Gudauskas G (1982) Rationale for the use of alternating non-cross-resistant chemotherapy. *Cancer Treatment Reports* **66**: 439–449

Glick JH, Young ML, Harrington D, Schilsky RL, Beck T, Neiman R, Fisher RI, Peterson B and Olsen MM (1998) MOPP/ABV Hybrid chemotherapy for advanced Hodgkin's Disease significantly improves failure-free and overall survival: The 8-year results of the Intergroup trial. *J Clin Oncol* **16**: 19–26

Hancock BW, Vaughan Hudson G, Vaughan Hudson B, Haybittle JL, Bennett MH, Maclennan KA and Jelliffe AM (1991) British National Lymphoma Investigation randomised study of MOPP (mustine, Oncovin, procarbazine, prednisolone) against LOPP (Leukeran substituted for mustine) in advanced Hodgkin's disease – long term results. *Br J Cancer* **63**: 579–582

Hancock BW, Vaughan Hudson B, Vaughan Hudson G, Bennett MH, Maclennan KA, Haybittle JL, Anderson L and Linch DC (1992) LOPP alternating with EVAP is superior to LOPP alone in the initial treatment of advanced Hodgkin's disease: results of a British National Lymphoma Investigation trial. *J Clin Oncol* **10**: 1252–1258

Kaplan EL and Meier P (1958) Nonparametric estimation from incomplete observations. *American Statistical Association Journal* **53**: 457–481

Linch DC and Vaughan Hudson B (1988) Management of the malignant lymphomas. In: Hoffbrand AV (ed) *Recent Advances in Haematology* London: Longman Group pp 211–242

Longo DL, Young RC, Wesley M, Hubbard SM, Duffey PL, Jaffe ES and De Vita VT Jr (1986) 20 years of MOPP therapy for Hodgkin's disease. *J Clin Oncol* **4**: 1295–1306

Longo DL, Duffey PL, De Vita VT, Wiernik PH, Hubbard SM, Phares JC, Bastian AW, Jaffe ES and Young RC (1991) Treatment of advanced-stage Hodgkin's disease: alternating non-cross resistant MOPP/CABS is not superior to MOPP. *J Clin Oncol* **8**: 1409–1420

Loeffler M, Brosteanu O, Hasenclever D, Sextro M, Assouline D, Bartolucci AA, Cassileth PA, Crowther D, Diehl V, Fisher RI, Hoppe RT, Jacobs P, Pater JL, Pavlovsky S, Thompson E and Wiernik P (1998) Meta-analysis of chemotherapy versus combined modality treatment trials in Hodgkin's disease. *J Clini Oncol* **16**: 818–829

Nicholson WM, Beard MEJ, Crowther D, Stansfeld AG, Vartan CP, Malpas JS, Hamilton Fairley G and Bodley Scott R (1970) Combination chemotherapy in generalised Hodgkin's disease. *BMJ* **3**: 7–10

Peto R, Pike MC, Armitage P, Breslow NE, Cox DR, Howard SV, Mantel N, McPherson K, Peto J and Smith PG (1977) Design and analysis of randomised clinical trials requiring prolonged observation of each patient. II. analysis and examples. *Br J Cancer* **35**: 1–39

Radford JA, Crowther D, Rohatiner AZS, Ryder WDJ, Gupta RK, Oza A, Deakin DP, Arnott S, Wilkinson PM, James RD, Johnson RJ and Lister TA (1995) Results of a randomised trial comparing MVPP chemotherapy with a hybrid regimen, ChlVPP/EVA, in the initial treatment of Hodgkin's disease. *J Clin Oncol* **13**: 2379–2385

Santoro A, Bonadonna G, Bonfante V and Valagussa P (1982) Alternating drug combinations in the treatment of advanced Hodgkin's disease. *New Engl J Med* **306**: 770–775

Selby P, Patel P, Milan S, Meldrum M, Mansi J, Mbidde E, Brada M, Perren T, Forgeson G, Gore M, Smith I and McElwain T (1990) ChlVPP combination chemotherapy for Hodgkin's disease; long-term results. *Br J Cancer* **62**: 279–285

Vinciguerra V, Propert KJ, Coleman M, Anderson JR, Stutzman L, Pajak TF, Nissen NI, Frizzera G, Gottleib A and Holland JF (1986) Alternating cycles of combination chemotherapy for patients with recurrent Hodgkin's disease following radiotherapy. A prospectively randomised study by the Cancer And Leukaemia Group B. *J Clin Oncol* **4**: 838–846

Wagstaff J, Gregory WM, Swindell R, Crowther D and Lister TA (1988) Prognostic factors for survival in stage IIIB and IV Hodgkins disease: A multivariate analysis comparing two specialist treatment centres. *Br J Cancer* **58**: 487–492

*© 2001 Cancer Research Campaign*
